# Well-Defined Diimine Copper(I) Complexes as Catalysts in Click Azide-Alkyne Cycloaddition Reactions

**DOI:** 10.3390/molecules18088919

**Published:** 2013-07-26

**Authors:** Jordi Markalain Barta, Silvia Díez-González

**Affiliations:** Department of Chemistry, Imperial College London, Exhibition Road, South Kensington, SW7 2AZ London, UK; E-Mail: j_markalain@hotmail.com

**Keywords:** ligand, diimine, copper(I), Click, dipolar cycloaddition, azide

## Abstract

A series of 1,4-disubstituted 1,2,3-triazoles have been prepared in high yields while respecting the stringent Click criteria. In these reactions, highly stable pre-formed complexes bearing diimine ligands were used.

## 1. Introduction

**Figure 1:**
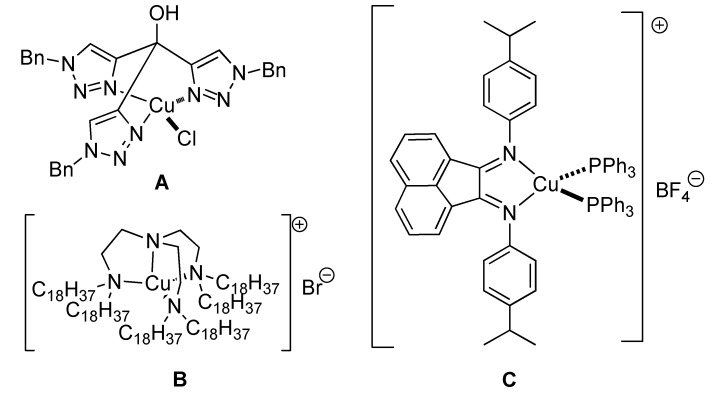
Reported preformed copper(I) catalysts with *N*-ligands.

The introduction of copper(I) catalysts in the cycloaddition of azides and terminal alkynes represents one of the latest success stories of organometallic catalysis [1,2,3,4]. Not only is this transformation high yielding and completely regioselective, but also it exemplifies the utility and importance of Click chemistry [5]. Whereas this cycloaddition reaction has been applied in a plethora of fields, the efforts to develop efficient catalytic systems, respectful of the Click criteria, have been significantly fewer. Nevertheless, the use of ligands in the cycloaddition of alkynes and azides has been shown to stabilize the copper(I) centre, increase the catalytic activity, and even modulate it [6]. Among the different families of ligands applied in this reaction, nitrogen-based ones are arguably the most widely used. Whereas Meldal *et al.* used diisopropylethylamine in their groundbreaking report [1], *N,N',N''*-pentametyletylentriamine (PMDETA) and tris-triazoles are also very popular choices [6]. However, examples of well-defined catalysts containing nitrogen based remain scarce [7,8,9,10,11]. Most reported examples employ polydentate ligands such as polytriazoles or tren ligands (Figure 1). Particularly relevant to this work are complexes bearing bis(aryl)acenaphthenenquinonediimine (Ar-BIAN) ligands **C**. These have been shown to lead to the formation of triazoles in conversion ranging from modest to good in THF at 50 °C [11]. 

**Figure 2:**
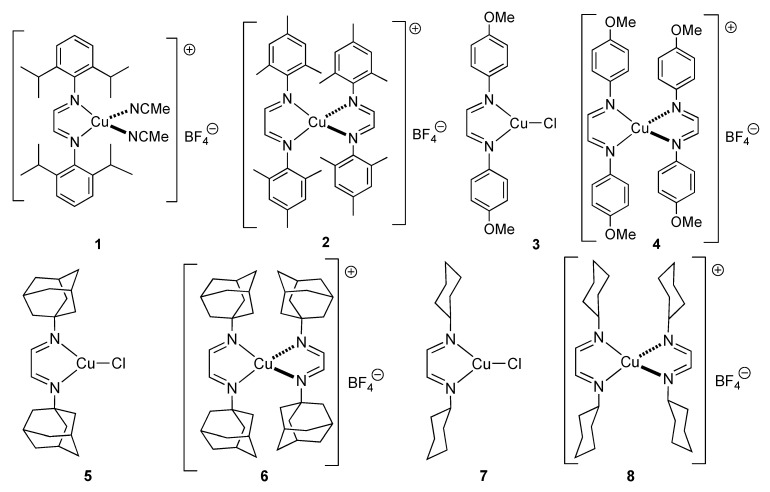
Copper(I) catalysts used in this study.

Herein, we report the catalytic activity of pre-formed copper(I) complexes bearing one or two α-diimine ligands in the preparation of 1,2,3-triazoles from azides and terminal alkynes. The structures of the screened complexes are shown in Figure 2. These complexes are all highly stable and easy to prepare from simple diazabutadiene compounds [12].

## 2. Results and Discussion

We started our optimization studies with the screening of different solvents. We chose cationic complex **1** and benzyl azide (**9a**) and phenylacetylene as the model reaction. Using 5 mol% of the copper catalyst, virtually no conversion of the starting materials was observed with MeCN, cyclohexane, or ethyl acetate, whereas poor results were obtained in MeOH or under neat conditions (Table 1). Satisfyingly, complete conversions into triazole **10a** were obtained on water and in acetone (Table 1, entries 8 and 9). Similar results were observed in THF or in a mixture water/*t*-BuOH, however, reactions in these solvents were not always reproducible and they were not studied further (Table 1, entries 6 and 7).

In order to determine the best reaction medium for this reaction, the copper loading was next reduced to 2 mol% with acetone and water (Table 1, entries 10 and 11). A higher conversion of 86% was observed in acetone and hence it was kept as solvent for the rest of the study. It is important to note that all tested solvents were technical grade, and in particular, the acetone employed here was the one normally used for cleaning the glassware in the laboratory.

**Table 1:** Solvent screening. 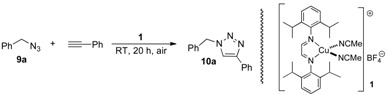

Entry	Solvent	[Cu] (mol%)	Conv. (%) ^a^
1	MeCN	5	<5
2	Cyclohexane	5	<5
3	Ethyl Acetate	5	<5
4	MeOH	5	52
5	Neat	5	13
6	THF	5	84 to <5
7	Water/*t*-BuOH	5	>95 to 9
8	Water	5	>95
9	Acetone	5	>95
10	Water	2	52
11	Acetone	2	86

^a^ ^1^H-NMR conversions are the average of at least two independent reactions.

**Scheme 1:**
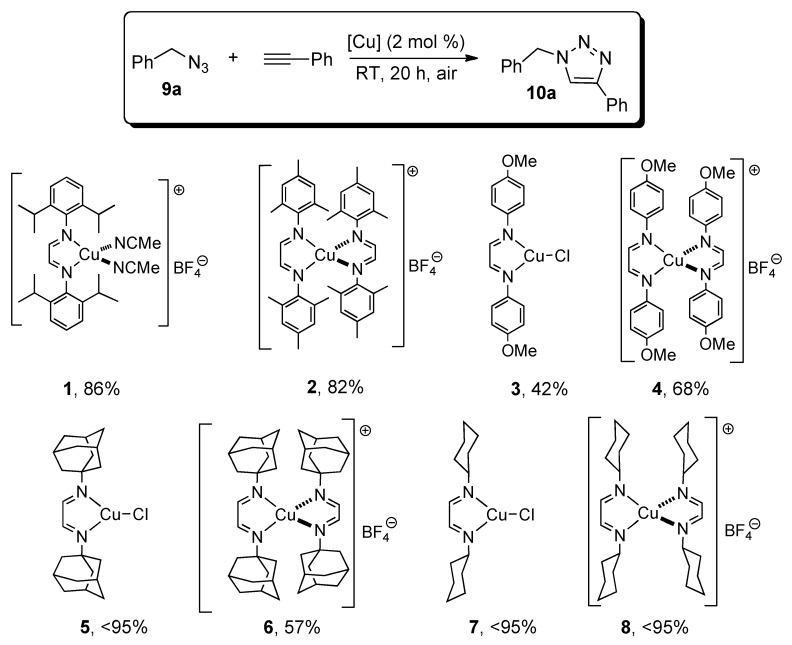
Catalyst screening ^a^.

^a^ ^1^H-NMR conversions are the average of at least two independent reactions.

We next performed a catalyst screening in acetone at room temperature using 2 mol% of different diimine copper complexes (Scheme 1). All the tested complexes performed well in the model reactions with conversion ranging from 42% to complete. For complexes bearing aromatic groups on the ligands, steric hindrance proved to be more beneficial to the reaction outcome than the presence of electron donating groups. In general, cationic complexes performed better than their neutral analogues, except for complexes bearing adamantyl imine ligands **5** and **6**. Additionally, the best performing complexes had aliphatic substituents on the diimine ligand(s). Hence, complexes **5**, **7** and **8** led to total conversions and further experiments were run with these in order to determine the best performing one.

First, the metal loading was further reduced (Table 2). Similar results were obtained with 1 mol% [Cu], but at 0.5 mol% [Cu] it became apparent that complexes bearing either one or two cyclohexyl diimine ligands **7** and **8** displayed a better activity than complex **5**, with adamantyl substituents. Gratifyingly, complex **8** could still achieve total conversion in a more concentrated reaction (1 M rather than 0.5 M), and it was chosen as the optimal catalyst within the tested series.

**Scheme 2:**
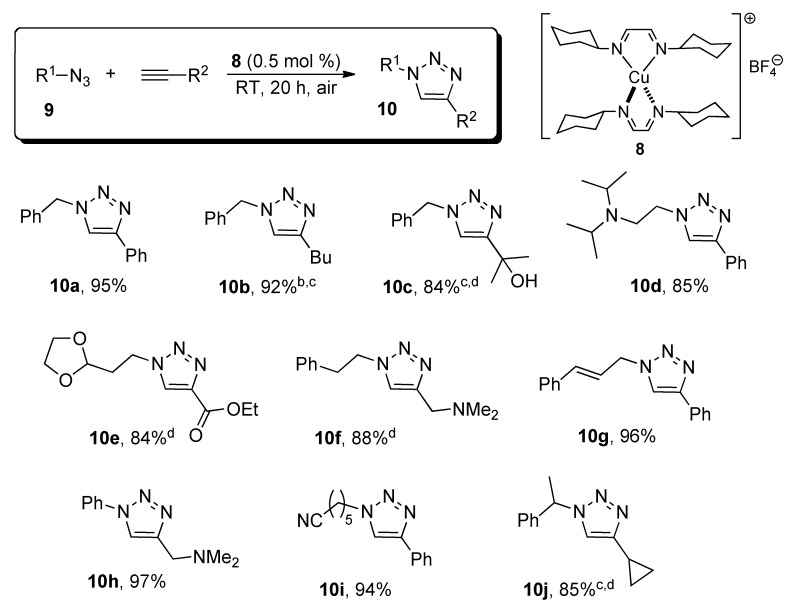
Preparation of 1,2,3-triazoles catalyzed by complex **8** ^a^.

^a^ Isolated yields are the average of at least two independent reactions; ^b^ 2 mol% **8**; ^c^ 40 °C; ^d^ 1 mol% **8**.

With an optimized catalytic system in hand, the scope of the reaction was then explored. All reactions were carried out in technical grade acetone, in air and in no cases oxidation to copper(II) or disproportionation to copper(0) and copper(II) were observed. Also, no other by-products were formed and the prepared triazoles could be easily isolated as pure products in high yields after a simple extraction (Scheme 2).

A number of functional groups were well tolerated, such as alcohols, amines, alkenes and nitriles. Benzyl, alkyl and aryl azides well suitable cycloaddition partners, as well as electron rich or electron poor alkynes. In some cases, total conversions were not reached under the optimized conditions. However, a slight increase in the reaction temperature (from RT to 40 °C) or the metal loading was enough to ensure a high isolated yield.

**Table 2:** Final optimization reactions. 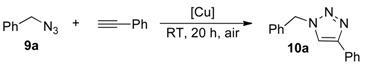

Entry	[Cu]	Concentration (M)	Conv. (%) ^a^
1	**5**, 1 mol%	0.5	<95
2	**7**, 1 mol%	0.5	<95
3	**8**, 1 mol%	0.5	<95
4	**5**, 0.5 mol%	0.5	63
5	**7**, 0.5 mol%	0.5	92
6	**8**, 0.5 mol%	0.5	<95
7	**7**, 0.5 mol%	1.0	93
8	**8**, 0.5 mol%	1.0	<95

^a^ ^1^H-NMR conversions are the average of at least two independent reactions.

## 3. Experimental Section

### 3.1. General

All reagents were commercially available and used as received. ^1^H-NMR (400 MHz) and ^13^C-NMR (100 MHz) spectra were recorded in CDCl_3_ on a Bruker AVANCE400 spectrometer at room temperature. Chemical shifts (δ) are reported in ppm and referenced to tetramethylsilane (^1^H) and deuterated chloroform (^13^C), respectively. All reactions were carried out in air and using technical solvents with no particular precautions to exclude oxygen or moisture. All reported yields are isolated yields and are the average of at least two independent reactions. Benzyl azide (**9a**) [13] 2-azidoethyl-diisopropylamine (**9b**) [14], 2-(2-azidoethyl)-1,3-dioxolane (**9c**) [15], (2-azidoethyl)benzene (**9d**) [13], 3-(azidoprop-1-en-yl)benzene (**9e**) [16], phenyl azide (**9f**) [17], 6-azidohexanenitrile (**9g**) [18] and (1-azidoethy)benzene (**9h**) [13] are known in the literature and the corresponding spectroscopic data for all these compounds were in good agreement with the reported data.

### 3.2. Catalytic Results

*General Procedure for the [3+2] Cycloaddition of Azides and Terminal Alkynes*

In a vial fitted with a screw cap, azide **9** (1 mmol), alkyne (1 mmol), acetone (1 mL) and **8** (3 mg, 0.5 mol%) were loaded. The reaction was allowed to proceed at room temperature overnight (20 h). The reaction mixture was then hydrolyzed with a saturated aqueous solution of NH_4_Cl (1 h) and extracted with EtOAc. In all examples, the crude products were estimated to be greater than 95% pure by ^1^H-NMRs.

*1-Benzyl-4-phenyl-1H-1,2,3-triazole* (**10a**). Using the general procedure 223 mg (95%) of the title compound were prepared from benzyl azide (**9a**, 125 μL) and phenylacetylene (112 μL). Spectroscopic data for **10a** were consistent with previously reported data for this compound [19]. ^1^H-NMR (CDCl_3_): δ 7.80 (d, *J* = 7.0 Hz, 2H, H_Ar_), 7.66 (s, 1H, NC*H* = C), 7.42–7.37 (m, 5H, H_Ar_), 7.34–7.31 (m, 3H, H_Ar_), 5.59 (s, 2H, PhC*H*_2_). ^13^C-NMR (CDCl_3_): δ 148.2 (C, =*C*–Ph), 134.7 (C, C_Ar_), 130.5 (C, C_Ar_), 129.1 (CH, CH_Ar_), 128.8 (CH, CH_Ar_), 128.8 (CH, CH_Ar_), 128.1 (CH, CH_Ar_), 128.0 (CH, CH_Ar_), 125.7 (CH, CH_Ar_), 119.5 (CH, N*C*H=), 54.2 (CH_2_).

*1-Benzyl-4-butyl-1H-1,2,3-triazole* (**10b**). Using the general procedure 199 mg (92%) of the title compound were prepared from benzyl azide (**9a**, 125 μL), 1-hexyne (115 μL), and **8** (12 mg, 2 mol%) at 40 °C, Spectroscopic data for **10b** were consistent with previously reported data for this compound [10]. ^1^H-NMR (CDCl_3_): δ 7.40–7.34 (m, 3H, H_Ar_), 7.26–7.24 (m, 2H, H_Ar_), 7.18 (s, 1H, NC*H*=C), 5.50 (s, 2H, PhC*H*_2_N), 2.69 (t, *J* = 7.5 Hz, 2H, C=CNC*H*_2_), 1.62 (quintet, *J* = 7.5 Hz, 2H, CH_2_C*H*_2_CH_2_), 1.37 (sextet, *J* = 7.3 Hz, 2H, C*H*_2_CH_3_), 0.91 (t, *J* = 7.3 Hz, 3H, CH_3_). ^13^C-NMR (CDCl_3_): δ 149.8 (C, NC=*C*-butyl), 135.0 (C, C_Ar_), 129.0 (CH, CH_Ar_), 128.6 (CH, CH_Ar_), 127.9 (CH, CH_Ar_), 120.9 (CH, NCH=), 54.0 (CH_2_, PhCH_2_), 31.4 (CH_2_), 25.4 (CH_2_), 22.3 (CH_2_), 13.8 (CH_3_).

*1-Benzyl-4-(2-hydroxypropan-2-yl)-1H-1,2,3-triazole* (**10c**). Using the general procedure 183 mg (84%) of the title compound were prepared from benzyl azide (**9a**, 125 μL), 2-methylbut-3-yn-2-ol (100 μL), and **8** (6 mg, 1 mol%) at 40 °C. Spectroscopic data for **10c** were consistent with previously reported data for this compound [19]. ^1^H-NMR (CDCl_3_): 7.39–7.35 (m, 3H, H_Ar_), 7.34 (s, 1H, NC*H*=C), 7.31–7.27 (m, 2H, H_Ar_), 5.51 (s, 2H, CH_2_), 2.34 (br s, 1H, OH), 1.61 (s, 6H, CH_3_). ^13^C-NMR (CDCl_3_): δ 156.0 (C, NC=*C*–), 134.6 (C, C_Ar_), 129.2 (CH, CH_Ar_), 128.6, (CH, CH_Ar_), 128.2 (CH, CH_Ar_), 119.1 (CH, NCH=), 68.3 (C, *C*(OH)Me_2_), 54.2 (CH_2_, PhCH_2_), 30.5 (CH_3_).

*1-(2-Diisopropylaminoethyl)-4-phenyl-1H-1,2,3-triazole* (**10d**). Using the general procedure 232 mg (85%) of the title compound were prepared from azide **9b** (170 mg) and phenylacetylene (112 μL). ^1^H-NMR (CDCl_3_): δ 7.84–7.81 (m, 3H, H_Ar_), 7.45 (t, *J* = 6.4 Hz, 2H, H_Ar_), 7.35 (t, *J* = 6.4 Hz, 1H, H_Ar_), 4.38 (t, *J* = 6.5 Hz, 2H, CH_2_), 3.04 (septet, *J* = 6.5 Hz, 2H, C*H*(CH_3_)_2_), 2.95 (t, *J* = 6.5 Hz, 2H, CH_2_), 0.99 (d, *J* = 6.5 Hz, 12H, CH_3_); ^13^C-NMR (CDCl_3_): δ 147.1 (NCH=*C*), 130.9 (C, C_Ar_), 128.8 (CH, CH_Ar_), 127.9 (CH, CH_Ar_), 125.7 (CH, CH_Ar_), 120.8 (CH, N*C*H=), 51.2 (CH_2_, *C*H_2_N), 48.7 (CH, *C*HN), 45.7 (CH_2_, *C*H_2_N), 20.8 (CH_3_). HRMS calculated for C_16_H_25_N_4_: 273.2079; found 273.2086 [(M+H)^+^].

*Ethyl 1-(2-(1,3-dioxolan-2-yl)ethyl)-1H-1,2,3-triazole-4-carboxylate* (**10e**). Using the general procedure 203 mg (84%) of the title compound were prepared from azide **9c** (143 mg), ethyl propiolate (102 μL), and **8** (6 mg, 1 mol%). Spectroscopic data for **10e** were consistent with previously reported data for this compound [20]. ^1^H-NMR (CDCl_3_): δ 8.12 (s, 1H, NC*H*=C), 4.93 (t, *J* = 4.0 Hz, 1H, OC*H*O), 4.58 (t, *J* = 7.0 Hz, 2H, CH_2_C*H*_2_N), 4.43 (q, *J* = 7.0 Hz, OC*H*_2_CH_3_), 4.03–3.93 (m, 2H, OCH_2_CH_2_O), 3.93–3.83 (m, 2H, OCH_2_CH_2_O), 2.33 (dt, *J* = 4.0; 7.0 Hz, CHC*H*_2_CH_2_), 1.41 (t, *J* = 7.0 Hz, 3H, OCH_2_C*H*_3_). ^13^C-NMR (CDCl_3_): δ 160.2 (C, C=O), 139.3 (C, =*C*–CO_2_Et), 127.5 (CH, CH=C), 127.5 (CH, O–CH–O), 64.55 (CH_2_, O–CH_2_–CH_2_–O), 64.48 (CH_2_, O–CH_2_–CH_2_–O), 60.6 (CH_2_, O*C*H_2_–CH_3_), 45.0 (CH_2_, CH_2_–*C*H_2_–N), 33.2 (CH_2_, *C*H_2_–CH_2_–N), 13.7 (CH_3_).

*N,N-Dimethyl-1-(1-phenethyl-1H-1,2,3-triazol-4-yl)methanamine* (**10f**). Using the general procedure 202 mg (88%) of the title compound were prepared from (2-azidoethyl)benzene (**9d**, 147 mg), dimethylprop-2-ynylamine (111 μL) and **8** (6 mg, 1 mol%). Spectroscopic data for **10f** were consistent with previously reported data for this compound [20]. ^1^H-NMR (CDCl_3_): δ 7.31–7.21 (m, 4H, H_Ar_ + NC*H*=C), 7.09 (d, *J* = 7.0 Hz, 2H, H_Ar_), 4.58 (t, *J* = 7.0 Hz, 2H, PhCH_2_C*H*_2_), 3.57 (s, 2H, C*H*_2_NMe_2_), 3.21 (t, *J* = 7.0 Hz, 2H, PhC*H*_2_CH_2_), 2.23 (s, 6H, NMe_2_). ^13^C-NMR (CDCl_3_): δ 144.7 (C, NCH=*C*), 137.0 (C, C_Ar_), 128.7 (CH, CH_Ar_), 128.6 (CH, CH_Ar_), 126.9 (CH, CH_Ar_), 122.6 (CH, N*C*H=), 54.2 (CH_2_, PhCH_2_C*H*_2_N), 51.4 (CH_2_, *C*H_2_NMe_2_), 44.9 (CH_2_, Ph*C*H_2_), 36.6 (CH_3_).

*4-Phenyl-1-(3-phenyl-2-propenyl)-1H-1,2,3-triazole* (**10g**). Using the general procedure 250 mg (96%) of the title compound were prepared from azide **9e** (159 mg) and phenylacetylene (112 μL). Spectroscopic data for **10g** were consistent with previously reported data for this compound [21]. ^1^H-NMR (CDCl_3_): δ 7.84–7.82 (m, 3H, H_Ar_ + NC*H*=C), 7.43–7.39 (m, 4H, H_Ar_), 7.36–7.32 (m, 4H, H_Ar_), 6.72 (d, *J* = 16.0 Hz, 1H, PhC*H*=CH), 6.40 (dt, *J* = 16.0, 6.5 Hz, 1H, PhCH=C*H*), 5.20 (d, *J* = 6.5 Hz, 2H, C*H*_2_N). ^13^C-NMR (CDCl_3_): δ 148.0 (C, NCH=*C*), 135.4 (C, C_Ar_), 135.2 (CH, CH_Ar_), 130.5 (C, C_Ar_), 128.7 (CH, CH_Ar_), 128.6 (CH, CH_Ar_), 128.4 (CH, CH_Ar_), 128.0 (CH, CH_Ar_), 126.6 (CH, Ph*C*H=), 125.6 (CH, PhCH=*C*H), 121.8 (CH, CH_Ar_), 119.3 (CH, N*C*H=), 52.3 (CH_2_, CH_2_N).

*Dimethyl(1-phenyl-1H-1,2,3-triazol-4-ylmethyl)amine* (**10h**). Using the general procedure 196 mg (97%) of the title compound were prepared from phenyl azide (**9f**, 107 μL) and dimethylprop-2-ynylamine (111 μL). Spectroscopic data for **10h** were consistent with previously reported data for this compound [22]. ^1^H-NMR (CDCl_3_): δ 7.95 (s, 1H, NC*H*=C), 7.74 (d, *J* = 8.0 Hz, 2H, H_Ar_), 7.53 (t, *J* = 8.0, 2H, H_Ar_), 7.43 (t, *J* = 8.0 Hz, 1H, H_Ar_), 3.71 (s, 2H, C*H*_2_NMe_2_), 2.34 (s, 6H, NMe_2_). ^13^C-NMR (CDCl_3_): δ 146.0 (C, NCH=*C*), 137.0 (C, C_Ar_), 129.6 (CH, CH_Ar_), 128.5 (CH, CH_Ar_), 120.4 (CH, NCH=), 120.3 (CH, CH_Ar_), 54.3 (CH_2_, CH_2_N), 45.2 (CH_3_).

*6-(4-Phenyl-1,2,3-triazol-1-yl)hexanenitrile* (**10i**). Using the general procedure 227 mg (94%) of the title compound were prepared from 6-azidohexanenitrile (**9g**, 138 mg) and phenylacetylene (112 μL). Spectroscopic data for **10i** were consistent with previously reported data for this compound [23]. ^1^H-NMR (CDCl_3_): δ 7.85–7.82 (m, 2H, H_Ar_), 7.76 (s, 1H, NC*H*=), 7.45–7.42 (m, 2H, H_Ar_), 7.36–7.31 (m, 1H, H_Ar_), 4.44 (t, *J* = 7.0 Hz, 2H, C*H*_2_N), 2.36 (t, *J* = 7.0 Hz, 2H, C*H*_2_CN), 2.08–1.97 (m, 2H,C*H*_2_), 1.77–1.67 (m, 2H,C*H*_2_), 1.59–1.48 (m, 2H, C*H*_2_). ^13^C-NMR (CDCl3): δ 147.8 (C, C=*C*–Ph), 130.5 (C, C_Ar_), 128.8 (CH, C_Ar_), 128.1 (CH, C_Ar_), 125.6 (CH, C_Ar_), 119.5 (CH, *C*H=C–Ph), 119.3 (C, CN), 49.8 (CH_2_, CH_2_–N), 29.5 (CH_2_), 25.5 (CH_2_), 24.7 (CH_2_), 17.0 (CH_2_).

*4-Cyclopropyl-1-(1-phenylethyl)-1H-1,2,3-triazole* (**10j**). Using the general procedure 183 mg (85%) of the title compound were prepared from azide **9h** (147 mg), ethynylcyclopropane (87 μL) and **8** (6 mg, 1 mol%) at 40 °C. Spectroscopic data for **10j** were consistent with previously reported data for this compound [23]. ^1^H-NMR (CDCl_3_): δ 7.40–7.28 (m, 3H, H_Ar_), 7.27–7.24 (m, 2H, H_Ar_), 7.11 (s, 1H, NC*H*=C), 5.76 (q, 1H, *J* = 7.0 Hz, PhCH), 1.95 (d, 3H, *J* = 7.0 Hz, CH_3_), 1.93–1.87 (m, 1H, CH_cyclopropyl_), 0.94–0.87 (m, 2H, CH_2_), 0.84–0.79 (m, 2H, CH_2_). ^13^C-NMR (CDCl_3_): δ 150.2 (C=C_cyclopropyl_), 140.1 (C, C_Ar_), 128.9 (CH, CH_Ar_), 128.3 (CH, CH_Ar_), 126.4 (CH, CH^Ar^), 118.3 (CH, N–*C*H=C), 58.9 (CH, PhCH), 21.2 (CH_3_), 7.6 (CH, CH_cyclopropyl_), 6.7 (CH_2_, CH_2_).

## 4. Conclusions

Diimine copper(I) complexes are highly efficient catalysts for the [3+2] cycloaddition of azides and terminal alkynes. Low copper loadings were enough to ensure high isolated yields of 1,2,3-triazoles at room temperature, in air and in technical grade acetone. Furthermore, purification by column chromatography was not necessary, and a simple extraction was enough to isolate pure triazoles. The reported catalysts are noticeably more active than related Ar-BIAN complexes. This might be attributed to two factors: (1) the presence of alkyl groups on the diimine ligands; which in these series systematically outperformed aryl diimines; or (2) the increased chemical stability of simple diimine scaffolds, in particular towards oxidation and reduction reactions.
